# Can field botany be effectively taught as a distance course? Experiences and reflections from the COVID-19 pandemic

**DOI:** 10.1093/aobpla/plab079

**Published:** 2021-12-23

**Authors:** Alistair G Auffret, Adam Ekholm, Aino Hämäläinen, Mats Jonsell, Carl Lehto, Michelle Nordkvist, Erik Öckinger, Peter Torstensson, Maria Viketoft, Göran Thor

**Affiliations:** Department of Ecology, Swedish University of Agricultural Sciences, 75007 Uppsala, Sweden

**Keywords:** Floristics, learning, online course, pedagogics, plant identification, teaching

## Abstract

The COVID-19 pandemic that started in 2020 forced a rapid change in university teaching, with large numbers of courses switching to distance learning with very little time for preparation. Courses involving many practical elements and field excursions required particular care if students were to fulfil planned learning outcomes. Here, we present our experiences in teaching field botany in 2020 and 2021. Using a range of methods and tools to introduce students to the subject, promote self-learning and reflection and give rapid and regular feedback, we were able to produce a course that allowed students to achieve the intended learning outcomes and that obtained similarly positive student evaluations to previous years. The course and its outcomes were further improved in 2021. We describe how we structured field botany as a distance course in order that we could give the best possible learning experience for the students. Finally, we reflect on how digital tools can aid teaching such subjects in the future, in a world where public knowledge of natural history is declining.

## Teaser

In an era of large-scale biodiversity change and reductions in basic knowledge of natural history, it is important that practical courses in subjects like field botany continue to be taught effectively. Forced by the COVID-19 pandemic to teach plant identification with no direct contact with students, we used a range of methods and tools to promote self-learning and reflection in students, and to facilitate rapid feedback by teachers. Here, we present our method for producing a course that allowed students to achieve the intended learning outcomes and that obtained positive student evaluations.

## Introduction

Plants make up the largest fraction of living biomass on Earth (Bar-On *et al.* 2018), providing many of the biotic and abiotic conditions necessary for species of other taxonomic groups across ecosystems. As a consequence, the diversity of plant communities is a strong predictor of biodiversity in other taxa (Basset *et al.* 2012; Prober *et al.* 2015; Brunbjerg *et al.* 2018), as well of important ecosystem functions and services (Isbell *et al.* 2011; Hautier *et al.* 2018). Nonetheless, academics and conservationists are concerned about an apparent decline in the interest in and knowledge of plants and of botany in general among their colleagues and the general public (Balding and Williams 2016; Crisci *et al.* 2020). Potentially contributing to, or compounding the problem, is the reduction in opportunities to study natural history in higher education programmes (Tewksbury *et al.* 2014).

In 2020, the COVID-19 pandemic forced university teachers across the globe to quickly adapt their courses and teaching methods, and learn new skills in order to give courses online. Because face-to-face teaching was expected to be a strong driver of transmission (Brooks-Pollock *et al.* 2021), highly practical courses based in the lab and field were cancelled or postponed, or otherwise reorganized so that fewer students were met at a time—something that resulted in large increases in contact times for the teachers themselves. In Sweden, the Public Health Agency of Sweden recommended on 17 March 2020 that all universities should immediately switch to distance learning (https://www.folkhalsomyndigheten.se/nyheter-och-press/nyhetsarkiv/2020/mars/larosaten-och-gymnasieskolor-uppmanas-nu-att-bedriva-distansundervisning/). At that juncture, the university board at Swedish University of Agricultural Sciences (SLU) implemented these recommendations until at least the middle of June 2020. Therefore, despite the long history of botanical excursions taking place in and around Uppsala, Sweden since Linnaeus in the 1700s, our courses in field botany, along with all other teaching at the university would need to be taught as full-time distance courses.

Teaching students to identify plant species as a distance course presented a large challenge, but if done successfully, it could increase the potential for offering field botany courses in new formats, which might be a way of helping to stem the decline of natural history teaching at a time where such knowledge is increasingly important. In this paper, we describe our experiences from the unusual summers of 2020 and 2021. In 2020, we were to our knowledge the only university in Sweden to offer field botany courses completely without any face-to-face teaching, while in 2021 with slightly lighter restrictions, we were able to meet small groups of students and offer a successful hybrid course.

## The Traditional Method

The Department of Ecology at the Uppsala campus of SLU organizes four undergraduate courses in field botany each year, to students in agronomy (17 students in 2020), landscape architecture (61 students 2020), landscape engineering (27 students 2020) and biology (10 students 2020). Because each course is part of a degree programme and not available to independent students, the pandemic did not affect the number of students on the courses. The four courses have (and in 2020–21 had) similar intended learning outcomes and a similar structure, but slightly different contents according to the curricula of the respective degree programmes. For simplicity, we focus here on the course for landscape architects, which is the largest course in terms of number of students. The intended learning outcomes are:

*To learn to recognize the most common plants in the wild. After completing the course the student should be able to: Identify common wild plant species on the basis of their appearance, growth habit and environment, and identify, for nature conservation, habitats and species worthy of protection.*

The course consists of introductory lectures describing the course and societal need for species identification skills, an introduction to invasive species and the six groups of species of conservation value, according to various national and international directives and programmes (red-listed species, legally protected species, indicator species, keystone species, species of special responsibility and typical species). Students are also taught how species can be used in sustainable landscape (habitat) planning, and are given an introduction to 14 important plant families. The majority of teaching is delivered through the approximately eight full-day field excursions to different environments that are spread over a 3-week period. During these excursions, each teacher leads a group of approximately 15–20 students, presenting species in the visited habitat and demonstrating characteristics that are important for identifying them. Each course starts with a longlist of approximately 230 plant species that we plan to show the students, of which approximately 200 are seen by all students across the excursions. Keying plant species using the flora (Krok and Almquist 2013) is also taught and practised.

Examination takes the form of a walk where students are asked to identify 30 of the plant species seen by all students during the course, plus a lab-based section where students should identify 10 additional species (not found in habitats around the university campus). A full point is given for the correct binomial scientific name, with half a point given for a correct Swedish name or correct scientific genus. In addition to the species identification, students are asked to key out four species that were not on the course list, with four points given for each correct answer. Students are allowed to use the course flora (Krok and Almquist 2013) throughout the exam. Out of a possible 56 points (30 + 10 species identification, plus 16 for the keying exercise), students must score 33.5 points to pass. A mock test (walk only) earlier in the course is used to prepare the students for the exam, with a good performance resulting in up to three bonus points available for the final test, in order to promote steady learning throughout the course period.

A project where students are asked to put their botanical skills into practice is also part of the assessment. In small groups (4–8 students), they perform an inventory of a small area of anthropogenically influenced land (e.g. parkland or a cemetery). As well as uploading their observations to the Swedish species portal (https://www.artportalen.se/), each group prepares a short presentation where on the final day of the course, they show their findings and discuss potential improvements for sustainable habitat management.

## The 2020 Method

The change in circumstances brought about by the pandemic required us to think of alternative ways to allow students to achieve the intended learning outcomes. Although the outcomes are not specific regarding the number of species that should be recognized, our aim with the course in 2020 was to provide an as similar as possible level of plant identification knowledge as the traditional course. In previous years, there had been a lot of contact between teachers and students during full-day excursions, with ample opportunity for students to ask questions and for teachers to identify students who may have needed more help. In 2020, however, it was necessary that the students would take a greater control of their learning, including—importantly—reflecting upon their own progress towards meeting the learning outcomes. As such, the course teaching became more characteristic of the ‘constructive alignment’ framework of teaching and learning (Biggs and Tang 2011), and it was our job as teachers to provide the means by which students could both learn the course material and evaluate their own learning.

**Figure 1.**
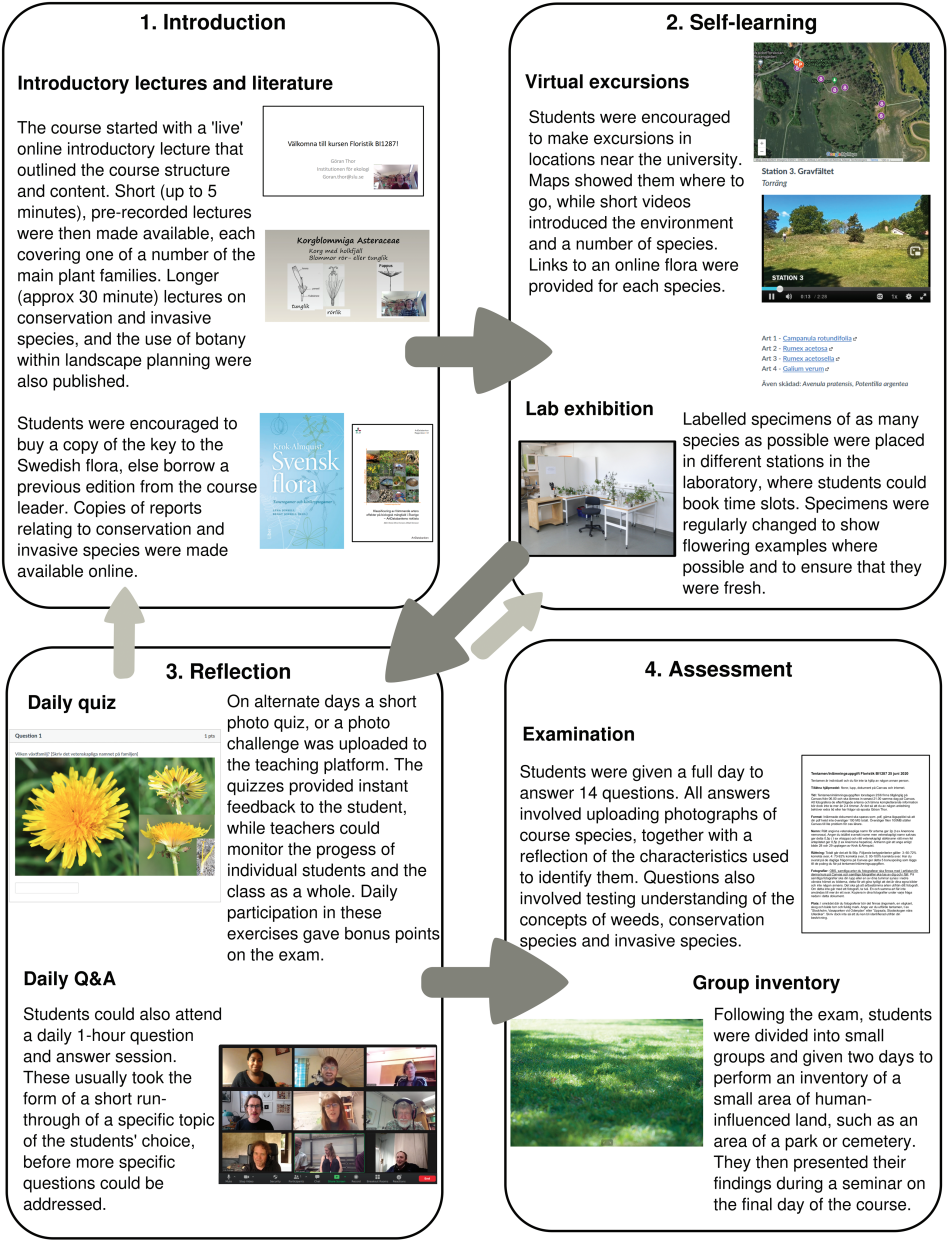
Outline of distance-learning method for field botany 2020. Students are given a background introduction in the subject before self-learning plant species identification using different methods. Continual reflection and self-assessment allows students to track and evaluate their learning, allowing them to revisit teaching materials as necessary. The examination allows students to show that they remember a number of plant species, describe and reflect on that knowledge. The group inventory gives an opportunity to apply the knowledge that they have gained.

We used a number of different teaching methods that come under four broad headings: (i) *Introduction* to the structure of the course, and basic knowledge of plants and the environments in which they grow; (ii) *Self-learning*, where we provided students with multiple ways in which they could learn to recognize the species on the course list; and (iii) *Reflection*, where students themselves or with the help of a teacher could test their learning, before going back to the introductory and self-learning material when necessary. Finally, (iv) *Assessment* involved the examination of learning outcomes. These stages are described in the text below, and illustrated in Fig. 1. The course material was hosted on the Canvas teaching platform (http://www.instructure.com), according to the contract with SLU.

### Introduction

If students are to be able to reflect upon their progress towards achieving a course’s intended learning outcomes, then it is important to inform the students of those outcomes. The introductory part of the course covered this, as well as introducing the key concepts that underpin *the identification of habitats and plant species* that forms the core goals of the course, something that was also included in the pre-pandemic version of the course.

#### Recorded lectures.

Aside from a ‘live’ online lecture introducing the course, all lectures were pre-recorded and made available on Canvas. The lectures were designed to give a broad introduction to the course content and a platform from which students could continue to work towards the learning outcomes by themselves. Lectures were typically divided into short recordings, e.g. the lecture on plant families was divided into fourteen 3- to 5-min recordings each presenting one family. This was both to avoid the need for students to concentrate during long online lectures, as well as to facilitate revisitation of specific topics, or plant families later in the course. All recorded lectures were available to the students for the whole duration of the course.

### Self learning

Once the students had been made aware of the intended learning outcomes, we provided two main ways that the students could gain the expected knowledge. These were designed so that students could learn to *recognize and identify common plants based on their appearance*, but also to make the explicit link between the plants and their environment.

#### Virtual excursions.

In order that students could complete the learning outcomes related to the *habitats and environments* in which species are found, we thought that it was important that students visit a range of habitats, even if teacher-led excursions were not possible. To this end, we created video excursions from six of the course’s traditional excursion sites. All six sites were easily reachable on foot or by bicycle from SLU’s Uppsala campus. Each excursion consisted of a number of ‘stations’, where the local habitat was filmed, and around four plant species were presented and their identifying features described (a short example video is provided in the **Supporting Information—Appendix S2**). In total, 134 species were described on the excursion videos. For each excursion, a page on the course’s Canvas page contained a Google map showing the locations of each station (plus parking spaces and public toilets if available), together with a separate video from each station. Links to an online botany resource (*Den Virtuella Floran*: http://linnaeus.nrm.se/flora/) for all the species described at each station were provided so that students could look at more photographs of each species and read an alternative description of their appearance and habitat. Students were expected to visit each of the sites and study the plant species individually, guided by the recorded videos. To facilitate the use of the videos during these field visits, we produced low-resolution videos that students could watch in the field on their smartphones, in addition to the high-resolution videos available on Canvas. While in the field, students were also encouraged to look at other species in their surroundings. We suggested the use of the mobile app Pl@ntNet, which had been recommended as the best option for the Swedish flora (Aronsson 2019), although a more thorough investigation using the British flora suggests that while Pl@ntNet performs relatively well, there may be better options (Jones 2020).

#### Laboratory exhibition.

To give the students an additional opportunity to learn to identify the species in the course list, we also set up an exhibition using some of the department’s laboratory and greenhouse space. Having labelled specimens of the different species acted as a backup for the possibility that students could not locate specific species during their own excursions, and allowed us to fill in the gaps of the species on the course list that were not shown on the excursion videos. It also allowed the students to see species in flower that had not reached that stage on the excursion videos, with another advantage being that students can directly study and compare similar species that do not always grow together in the field. Although such exhibitions—where examples of plants are collected and displayed for students in a laboratory or greenhouse—are commonplace in many botany courses, this has not been feasible at SLU in recent years due to the high costs of room bookings.

For the duration of the course, students had access to the lab and greenhouse where plant specimens were exhibited. In the exhibition space, plant species were presented and labelled in seven stations that were arranged following the taxonomic structure of the course flora, each containing approximately 25–30 different species. In order to follow the requirement of distance learning, teachers were never present at the same time as students. Instead, the greenhouse was visited daily by a teacher before the room was opened for students, to ensure that the plants were still labelled correctly and to replace withered specimens. To guarantee social distancing among the students, the stations were physically spaced and shielded from each other, and students were required to sign up for hourly timeslots at each station using a simple online spreadsheet. In all, 163 species were displayed in the exhibition during the course, making a final total of 187 species across both the exhibition and the virtual excursions.

### Reflection

One of the main advantages of teaching botany face to face in the field is the regular flow of communication and feedback between teachers and their students. Students are able to ask questions or request clarification regarding how to identify certain species or use the key in the flora, while teachers can actively and passively gain an understanding of the students’ progress. When teaching field botany as a distance course, we introduced two main ways in which students could each day gauge the progress of their learning in relation to the intended learning outcomes, and get extra help where needed. As always, students were also able to e-mail the teachers at any time.

#### Daily photo quiz.

Students were encouraged to test their learning on a daily photo quiz on Canvas. Two types of quiz were provided on alternate days: (i) approximately five photographs of species were provided that the students should identify, and (ii) students were asked to upload photographs from the field of particular species or species of particular families. The first type of quiz tested the students’ ability to *recognize and identify common plants based on their appearance*, while the second required that they could also find species in their specific *habit or environment.* Correct answers to the former were provided instantly through Canvas’ *Quiz* function, while for the latter the uploaded photos were checked and feedback given if necessary. There were no consequences for students getting answers wrong, but all students who took part in each daily quiz were given five bonus points for the final exam. To ensure that every student was able to complete the quizzes, each quiz was available from 5 am on the day of the quiz until 9 pm the following weekday.

#### Daily online question and answer sessions.

Every weekday afternoon, there was a 1-h video meeting where students could ask questions and get help with different aspects of the course. Popular topics included identification help with grasses and sedges, something that students often find most difficult. Some sessions also involved practising keying out species using photographs that the teacher had prepared.

### Assessment

#### Examination.

For the examination, students were given an exam paper with 14 questions, requiring them to photograph specific species of particular families or species group (e.g. invasive species, species of nature conservation value or weeds) from the course’s final species list and to describe characteristics of the species or families that help in their identification. Students were given one (specific) day to complete the exam, with the exam becoming available at 6 am and submissions closing at 9 pm. Students were expected to work alone, but were allowed to use any course or other material in order to answer the questions. To avoid that pictures were downloaded from internet, they should include their hand-lens or one of their thumbs in the lower left corner of the photograph. Before the examination, we recommended that students should find a suitable area in which to complete their exam in advance. In order that the area would contain many of the species on the course list, it should include a patch of forest (with both coniferous and deciduous trees), a roadside and a meadow. Students were free to perform the exam anywhere in Sweden, and many or most of them performed their exams outside Uppsala. An example exam was available to the students for the week leading up to the exam so that they would know what to expect. The exam gave a total of 56 points, with 33.5 (60 %) required for the student to pass. Five bonus points were given to those students who completed every daily quiz. This was an increase from the three points for the mock test in previous years, designed to encourage self-reflection and incentivize learning. A translated version of the exam paper is available in the **Supporting Information—Appendix S1**. While quite different to the pre-pandemic examination, this method was similarly aligned to the intended learning outcomes in requiring students to *recognize and identify common plants based on their appearance*, while the relationship of each species to its *habitat and environment* was now incorporated through the student’s need to locate the species to photograph, instead of previous years’ walks where students would look at the environment where the plants were growing to aid in their identification. The 2020 examination also required students to formulate their own basis for identification, rather than only identifying the species.

#### Group inventory.

The group inventory task was included as in previous years. According to the rules set down by the university, the students were allowed to work together in small groups in the field, although we did encourage adherence to social-distancing recommendations. The final seminar in which groups presented their work took place online.

## Outcomes

### Results from 2020

The number of species covered through the virtual excursions and the lab exhibition (187) was at the lower end of the species usually seen in the course, but still broadly comparable to the approximately 200 species covered in normal years. Uptake of the activities offered in the course were generally high, but variable among students. According to the information provided by Canvas, students spent a mean ± SD 33 ± 38 h on the course website. Students completed on average 10 of the 11 daily quizzes, with 82 % of students participating in all 11. Use of the lab exhibition appeared lower, with 343 1-h bookings made across the seven stations (all containing different species) by the 105 students across the four courses (more detailed information not available). Question-and-answer sessions were well-attended.

**Figure 2.**
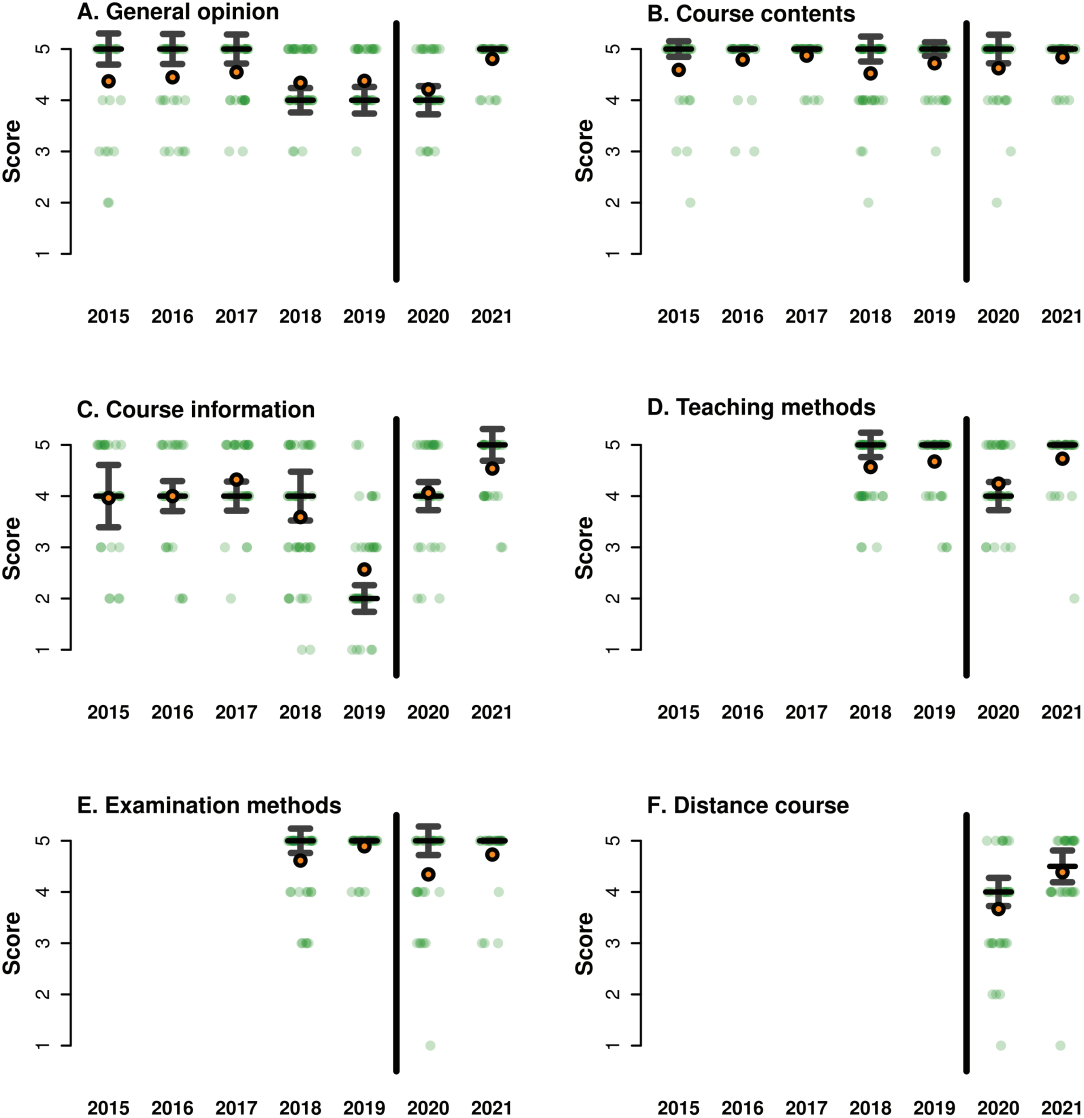
Comparison of anonymous course evaluations 2015–21. Translucent green points represent the individual answers to the question, horizontal black bars show the median and vertical grey bars show 95 % confidence intervals around the median. Solid orange points represent mean values. All answers were on a scale of 1–5, with 5 being the most positive. Full questions and answers to panels A–E are given in Table 1. Question F reads: My general opinion about the course being given virtually is 1=very poor – 5=very good.

Looking at pass rates, in 2020 98 % of the students passed the course, which is the modal pass rate from 2015 to 2019 (mean: 95.5 %). However, it is of course difficult to directly compare the very different types of examination, with the traditional course requiring students to memorize the species identifications. Hence, it is not possible to know how much students actually learned compared to previous years. Student evaluations of the course were overwhelmingly positive (Fig. 2). Despite a clear disappointment that the course could not be held with teacher-led field excursions, students were happy with the course organization and the teaching methods and materials that were used. All the different teaching material and methods (lectures, virtual excursions, lab exhibition, quizzes and Q&A sessions) were singled out by students in their free-text comments as being useful for their learning.

### 2021: a socially distanced hybrid course

In 2021, there were still strong restrictions on face-to-face teaching in Sweden, although the university ruled that we were able to give field excursions with up to nine students per group. Budget restrictions, and the availability of qualified teaching staff meant that it was not possible to teach full-day excursions to all 122 students across the four courses (although other Swedish universities recruited external teachers for their courses in 2021, K. Hylander, Stockholm University, pers. comm.). We also wanted to respect the students that would rather not attend in-person excursions, or otherwise could not, due to being elsewhere geographically following distance learning for the preceding term. This meant that the course was organized as in 2020, but with the additional option for students to sign up for and attend nine short (~3 h), local excursions in small groups, and with fewer online Q&A sessions. Another addition was that we also provided individual videos for each species from the virtual excursions, which were gradually complemented with additional species videos for as the course continued and more species were in flower. In total, 85 % of the species on the course longlist were filmed and uploaded by the time of the examination. Finally, a daily ‘plant of the day’ was posted on the Canvas page, giving the students an opportunity to learn or revise some important species and learn additional information such as historical uses for the plant, which can be useful mnemonic devices when recognizing species.

The excursions that were offered were well-attended, usually by around 70–80 % of the enrolled students, and were often mentioned in the free-text answers of the course evaluations as an important part of the course. However, those who did not attend the teacher-led excursions also appreciated having the possibility to go by themselves. The online resources were again well-used, with students spending 39 ± 37 h on the course website, and participating in 8.6 ± 2.7 of the 10 available daily quizzes (one fewer than 2020 due to how the course was scheduled). Visits to the laboratory exhibition were again relatively low, with 240 bookings across the seven stations made by the 122 students. The likely lower uptake of this resource in 2021 could be due to students generally taking advantage of the teacher-led excursions. Course evaluations again showed positive response to the course. In addition to the excursions, the availability of videos for individual species, as well as the plant of the day were also highlighted as valued tools that aided student learning in the student evaluations. The pass rate in 2021 was 94 %.

### Comparisons of student evaluations

Course evaluations in both 2020 and 2021 showed that student satisfaction was comfortably at the level of pre-COVID iterations of the course (Fig. 1). To address this statistically, we used two-sample Wilcoxon tests (also known as Mann–Whitney tests) to compare student evaluation scores for five main questions before (2015–19) and after (2020–21) the switch to online learning. These confirmed that as well as having comparable pass rates, the distance versions of the course also maintained similar levels of student satisfaction (Table 1). The only question where there was a significant difference across groups was for course information, where the switch to online learning actually resulted in higher satisfaction. This is likely due to both the relatively low score in 2018 when Canvas was first introduced, and the higher scores during the pandemic when a lot of effort was spent optimizing the platform (Fig. 2).

**Table 1.** Comparison of anonymous student evaluations for relevant questions (translated to English by the authors) regarding how the course was given before (2015–19, mean response rate 63 %), or after (2020–21, mean response rate 54 %) the switch to an online distance course.

	2015–19 (*n* = 168)		2020–21 (*n* = 59)		Wilcoxon test	
	Median	IQR	Median	IQR	*W*-statistic	*P*-value
My general opinion of the course was… 1 = Very poor to 5 = Very good	5	4–5	5	4–5	5158	0.6
The course content was clearly linked to the syllabus… 1 = Not at all to 5 = Completely	5	5–5	5	5–5	4786	0.79
The course information was easily accessible… 1 = Not at all to 5 = Completely	4	3–5	4	4–5	6449	0.0003
The course’s teaching methods have helped my learning*… 1 = Not at all to 5 = Completely	5	4–5	5	4–5	2112	0.17
The examination allowed me to show what I had learned during the course*… 1 = Not at all to 5 = Completely	5	5–5	5	4–5	2093	0.14

*Question was only asked from 2018 (*n* = 81 for 2018–19).

## Reflections for Future Years

We were very satisfied with how the course turned out in 2020. We believe that we successfully facilitated the students’ potential to reach the intended learning outcomes, and this was reflected in the examination results and the student evaluations. However, this success did come at a cost, with the course leader (GT) being required to spend >50 % extra time working out how to implement the course and considering different options amid often-changing recommendations and regulations. All teachers were involved in the course planning to a higher degree than in normal years, although for most teachers, the amount of time spent across the course was broadly comparable with previous years. This was because teachers were this time responsible for a different aspect of the course, rather than all leading excursion groups. The creation and editing of the excursion videos was also very time-consuming, and especially difficult given that less than a week passed between filming and the courses starting, to ensure as much as possible that plants were flowering in the videos.

The teaching load in 2021 was more comparable to previous years, with the 2020’s material (including lectures, quizzes and virtual excursions) and Canvas page structure already in place. Nonetheless, there was still a lot of planning work involved, with recommendations and regulations as well as COVID vaccination schedules changing regularly. The teaching load across the four courses was spread across more teachers than in previous years, in order that several groups could be taken on excursions, with other teachers only working behind the scenes with the exhibition and digital aspects of the course.

Although—global pandemic permitting—we are not planning to give field botany courses wholly as distance courses in the future, we are convinced that we will never completely go back to the previous course set-up. There are many aspects of the courses from 2020 to 2021 that both we and the students believe are extremely valuable and will be implemented into our regular courses in the future. In particular, we feel that providing a more varied range of activities for learning and reflection gives the student more agency in aligning their learning with the intended learning outcomes. Having short, recorded lectures available for students at all times was very useful for refreshing their memories about different plant families and for preparation for the examination. Online quizzes were a good way for students to test their knowledge in their own time, and could be particularly useful for those who do not feel comfortable answering direct questions in the field in front of their peers. It was also useful for teachers to be able to gauge how such quieter students are managing in learning the species. Videos describing each plant species can aid students’ revision of what has been learned, or can talk the student through a species that they are looking at in the lab or field.

It is clear that teacher-led group excursions are an important part of learning to identify plant species and their environments, as well as reflecting on their knowledge through feedback with the teacher. Group excursions also facilitates peer-based learning, in which students are able to talk to and help each other, something that is more difficult during a distance course. Another aspect of the courses that students found difficult was that the examination was found to be more time-consuming for the students than we teachers had anticipated. Nonetheless, this form of examination allowed students to carry out their exams anywhere in Sweden, and many of them choosing to do so outside Uppsala. Requiring students to formulate (and therefore think about) how they identified particular species, allows them to demonstrate a higher level of learning than simply remembering the names of plant species put in front of them. Importantly, we are convinced that the examination was an appropriate way of assessing the course’s learning outcomes.

A key area for improvement would be to teach the use of the species key in a better way, as it is an important skill for students in their future botanical learning. ‘Unknown’ species were left in the laboratory exhibitions for students to try to identify, and keying species was also taken up in the online Q&A sessions. However, we found it very difficult to do this without looking at the plants and the flora together with the students. In 2021, some time was spent keying species during the short excursions, but it was not examined.

In conclusion, we found that it was possible to successfully teach field botany as a distance course. Being forced to rethink how plant identification can be taught at short notice was a huge challenge, but we believe that it will improve teaching and learning in years to come. Although we think that nothing can really replace the experience of being in a habitat and looking at the plants together with the students, it feels possible that hybrid courses including a combination of distance learning and concentrated field-based teaching, for example as a residential activity, could be a way to reach more students in the future. This may then help to stem the ongoing and unfortunate loss of the knowledge of an interest in natural history.

## Supporting Information

The following additional information is available in the online version of this article—

**Appendix S1.** Examination paper from course in field botany at University of Agricultural Sciences 25 June 2020.

**Appendix S2.** Short example video from a virtual excursion.

**Appendix S3.** Student course evaluation scores 2015–2021.

plab079_suppl_Supplementary_Materials

## Data Availability

Summarized course evaluation responses from the course for field botany for landscape architects 2015–21 that we used for data analysis are available in the **Supporting Information**. SLU course evaluations are published online to aid prospective students, so evaluations of all field botany courses for recent years are available (in Swedish) at https://www.slu.se/utbildning/program-kurser/kurssok/?SearchQuery=floristik.

## Notes

**Plants, Ecosystems & Climate. Chief Editor**: Mary Heskel
