# Supplementary material for: Can field botany be effectively taught as a distance course? Experiences and reflections from the COVID-19 pandemic
Source: AoB Plants. 2021 Dec 23;14(1):plab079. doi: 10.1093/aobpla/plab079 (PMC8757578; doi:10.1093/aobpla/plab079)
Supplement: plab079_suppl_Supplementary_Materials [file plab079_suppl_supplementary_materials.zip › plab079_suppl_Supplementary_Appendix_S1.pdf]

Appendix S1
Examination paper from course in field botany at University of Agricultural Sciences 25 June 2020.
Translated from Swedish by Alistair Auffret.

This is an individual examination and you may not receive help from another person.

Permitted materials: Floras, handlens, online teaching material and the internet.

Time: The examination takes place 25/06/2020 and is available on Canvas from 06:00 and shall be submitted 21:00 at the latest. However, to photograph the required plants and add the required descriptions should not take more than 2-4 hours. If for any reason you need more time or have questions, please e-mail the course responsible.

Format: Submitted documents should be saved as .pdf files, preferably low-resolution so that they do not exceed 100mb.

Name: The correct scientific name for photographed species gives 2p (e.g. *Anemone nemorosa*). If a Swedish, but not scientific name is given, 0.5p is awarded (e.g. *Wood anenome*). Half a point is also awarded if the correct genus, but incorrect epithet is given (e.g. *Anemone hepatica*). Species names should match either the 28th or 29th editions of the Swedish Flora by Krok & Almquist.

Grading: There are a total of 56p available. The following criteria apply:
3: 60-72% correct answers; 4: 73-92% correct answers; 5: 93-100% correct answers. If you have answered the daily questions on Canvas, you will be awarded 5 additional bonus points.

Photographs: Note that all species that you photograph should be in the course's species list, and all photographs should be taken by you and in the field. Each picture should include your handlens or one of your thumbs in the bottom left corner, to make it clear that they are your own photographs and not anybody elses. It must be possible to identify the species from your photographs; if it is not possible with one picture, take two. Each species must only be used to answer one individual question. Import your photographs under each question in this document.

Location: The area where you carry out the exam should include grassland, a road verge, woodland and both dry and moist ground. State where you carried out the exam, e.g. "Vasaparken, Stockholm", or "Stadsskogen, near Ulleråker, Uppsala". Do not describe details that would identify you from other students.

1. Photograph a fern and a horsetail. Write which species they are and describe the characteristics you used to identify them. If you cannot find a species from each group, photograph two species from one of the groups. Where in the plant are the spores produced? (5p, 4p if two species of one group).

2. Photograph either common couch or perennial ryegrass. Write which species it is and describe the characteristics you used to identify it. How can you distinguish between the two species, and which one is considered a weed? If you cannot find either of these species, photograph another grass of choice and give its name. How can you recognise the grass family Poaceae? (4p, 2p if other grass species)

3. Photograph any two species of monocotyledons (except for grasses Poaceae). Write which species they are and describe the characteristics you used to identify them. (4p)

4. Photograph two species that are widely considered to be weeds, at least one of which should be from the daisy family (Asteraceae). If you cannot find an Asteraceae, photograph weeds of any family. Write which species they are and describe the characteristics you used to identify them. (5p, 4p if no Asteraceae)

5. Photograph one invasive species and one conservation species (specified in course list). Write which species they are and which species falls into which group. Describe the characteristics you used to identify them. (4p)

6. Photograph two species from the family Ranunculaceae, of which one should not have yellow flowers. Write which species they are and describe the characteristics you used to identify them. How do you distinguish between the buttercup family Ranunculaceae and the rose family Rosaceae? (5p)

7. Photograph two yellow-flowered species from the pea family Fabaceae. Write which species they are and describe the characteristics you used to identify them. How do you recognise the pea family? (5p)

8. Photograph a species of the genus Salix (Salicaceae). Write which species it is and describe the characteristics you used to identify it. If you cannot find a Salix, photograph and describe any other bush. (2p, 1p if not a Salix)

9. Photograph two species in the cabbage family Brassicaceae. Write which species they are and describe the characteristics you used to identify them. (4p)

10. Photograph a species of the genus Vaccinium (Ericaceae). Write which species it is and describe the characteristics you used to identify it. (2p)

11. Photograph two species from the plantain family Plantaginaceae. One should be from the genus Plantago and the other from the genus Veronica. If this is not possible, take two from the same genus. Write which species they are and describe the characteristics you used to identify them. (5p, 4p if both species are from the same genus)

12. Photograph two species from the daisy family Asteraceae, where at least one should have only ray- or disc flowers. Write which species they are, which types of flowers each species has and describe the characteristics you used to identify them. It is not necessary for the species to be flowering in the photographs. (5p)

13. Photograph any species from a family that you have not yet photographed. Write which species it is and describe the characteristics you used to identify it. (2p)

14. Photograph one moss and one lichen species. Write which species they are and describe the characteristics you used to identify them. (4p)
